# P-1104. Single-Dose and Repeat Single-Dose Ascending Dose Study Evaluating Safety, Tolerability, and Pharmacokinetics of Subcutaneous and Intramuscular CD388, a Novel Long-acting Drug-Fc Conjugate for Universal Prevention of Seasonal and Pandemic Influenza

**DOI:** 10.1093/ofid/ofae631.1292

**Published:** 2025-01-29

**Authors:** Shawn Flanagan, Ozlem Equils, Roxana E Rojas, Tristan Baguet, Sy-Shi Wang, Voon Ong, Taylor Sandison

**Affiliations:** Cidara Therapeutics, San Diego, CA; Cidara Therapeutics, San Diego, CA; Janssen Research & Development, Brisbane, California; Janssen R&D, Steenhuffel, Vlaams-Brabant, Belgium; Janssen Pharmaceutical, Brisbane, California; Cidara Therapeutics, San Diego, CA; Cidara Therapeutics, San Diego, CA

## Abstract

**Background:**

CD388 is a novel antiviral drug-Fc conjugate (DFC) designed to deliver universal prevention of seasonal and pandemic influenza. Nonclinical studies have shown CD388 was effective prophylactically against a wide range of seasonal and pandemic influenza A and B strains in lethal and nonlethal mouse models of infection. Interim clinical pharmacokinetics (PK), indicating similar intramuscular (IM) and subcutaneous (SQ) exposures and long half-life (t_1/2_), and blinded safety results, indicating no safety concerns with CD388 or placebo, were presented last year (ID Week 2023), and final results are now available.

**Figure 1:**
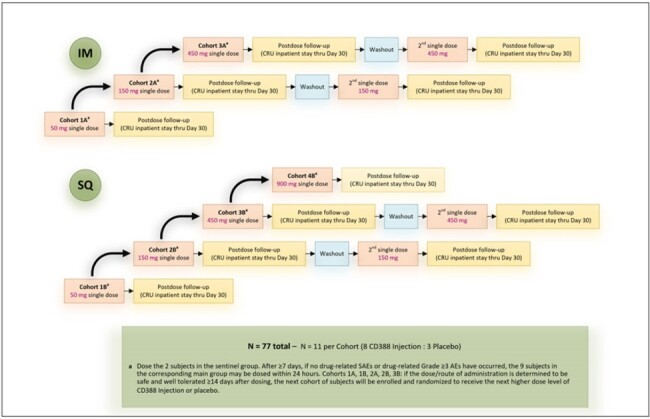
Design of CD388 First in Human Study

**Methods:**

The design of the CD388 First in Human study, conducted in healthy subjects is shown in Figure 1. CD388 for injection (100 mg/mL) was administered IM or SQ at doses of 50, 150, and 450, and 900 mg SQ only. Subjects in the 150 or 450 mg dose groups received a second single dose after a washout period of approximately 5 t_1/2_’s. Plasma CD388 concentrations and anti-CD388 antibody analysis were determined for subjects randomized to receive CD388 using validated methods. Safety was monitored throughout the study.

**Figure 2:**
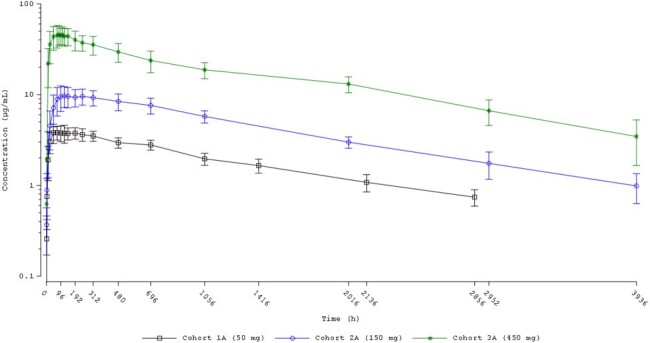
Single Dose Mean +/- SD Plasma CD388 Concentrations Versus Time for CD388 Administered by Intramuscular Injection (Semi-logarithmic Scale)

**Results:**

Pharmacokinetics of CD388 were similar following intramuscular or subcutaneous routes of administration. CD388 was absorbed rapidly and eliminated slowly with mean apparent half-life of elimination values ranging from ∼ 6 to 8 weeks (Figures 2 and 3). Increases in CD388 exposures were approximately dose proportional (Figure 4). Exposures were similar following a second single dose administration. Formation of anti-drug antibodies (ADA) were rare, at low titers when present, and did not affect PK.

Most treatment emergent adverse events (TEAEs) were mild in intensity, and there were no deaths, or serious AEs. Overall, no dose-dependent trend was observed for TEAEs, and the proportion of participants that experienced at least 1 TEAE or drug-related TEAE was similar for both administration routes (IM and SQ).

**Figure 3:**
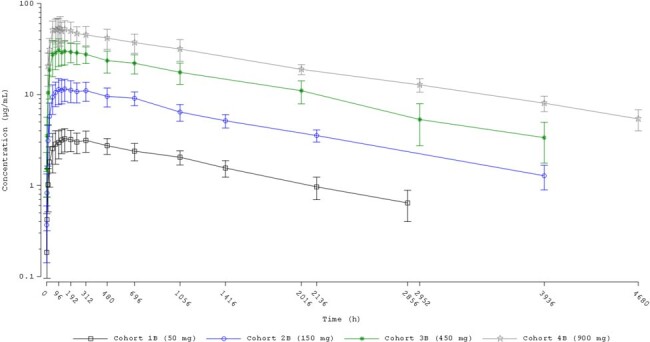
Single Dose Mean +/- SD Plasma CD388 Concentrations Versus Time for CD388 Administered by Subcutaneous Injection (Semi-logarithmic Scale)

**Conclusion:**

CD388 absorption by both routes was rapid and elimination was slow, indicating that seasonal influenza prevention could be achieved with one dose per season. Lack of significant ADA formation with repeat administration supports annual use. No safety concerns were noted in this study.

**Figure 4:**
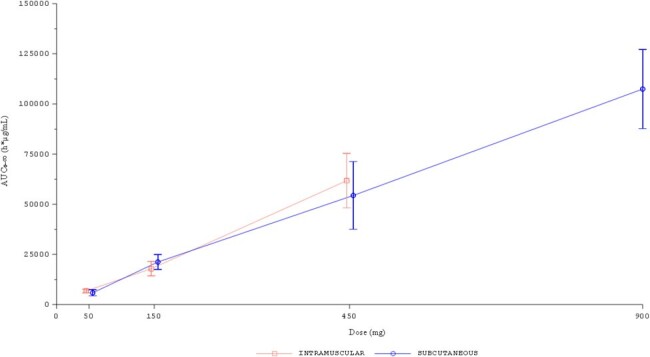
Single Dose Mean Plasma CD388 AUC for CD388 Administered by Intramuscular or Subcutaneous Injection

**Disclosures:**

**Shawn Flanagan, PhD**, Cidara Therapeutics: Salaried Employee|Cidara Therapeutics: Employee|Cidara Therapeutics: Stocks/Bonds (Public Company)|Cidara Therapeutics: Stocks/Bonds (Public Company) **Ozlem Equils, MD**, Cidara Therapeutics: Employee|Cidara Therapeutics: Stocks/Bonds (Public Company) **Roxana E. Rojas, M.D., Ph.D.**, Janssen Research & Development LLC: Employee|Janssen Research & Development LLC: Stocks/Bonds (Public Company)|Johnson and Johnson: Stocks/Bonds (Public Company) **Tristan Baguet, PharmD**, Johnson&johnson: Stocks/Bonds (Public Company) **Sy-Shi Wang, Ph.D.**, Janssen Research & Development LLC: Employee|Janssen Research & Development LLC: Stocks/Bonds (Public Company) **Voon Ong, Executive Director**, Cidara Therapeutics: Stocks/Bonds (Public Company) **Taylor Sandison, MD, MPH**, Cidara Therapeutics Inc: Employee|Cidara Therapeutics Inc: Stocks/Bonds (Public Company)

